# *Lactobacillus plantarum* KSFY06 and geniposide counteract montmorillonite‐induced constipation in Kunming mice

**DOI:** 10.1002/fsn3.1814

**Published:** 2020-08-07

**Authors:** Yi Gan, Jie Liang, Wenjing Diao, Xianrong Zhou, Jianfei Mu, Liang Pang, Fang Tan, Xin Zhao

**Affiliations:** ^1^ Chongqing Collaborative Innovation Center for Functional Food Chongqing University of Education Chongqing China; ^2^ Chongqing Engineering Research Center of Functional Food Chongqing University of Education Chongqing China; ^3^ Chongqing Engineering Laboratory for Research and Development of Functional Food Chongqing University of Education Chongqing China; ^4^ Department of Pediatrics Chongqing Traditional Chinese Medicine Hospital Chongqing China; ^5^ Department of Oral and Maxillofacial Surgery The Affiliated Hospital of Stomatology Chongqing Medical University Chongqing China; ^6^ Department of Public Health Our Lady of Fatima University Valenzuela Philippines

**Keywords:** constipation, geniposide, *Lactobacillus plantarum*, montmorillonite, mRNA

## Abstract

Constipation is a common clinical manifestation of digestive system disorders and occurs worldwide. This study investigated the ability of *Lactobacillus plantarum* KSFY06 (LP‐KSFY06) to promote the action of geniposide in preventing montmorillonite‐induced constipation in Kunming mice, with the aim of providing a successful solution. The effects of LP‐KSFY06 and geniposide on constipation were measured, and the results showed that the protective effect of geniposide on constipation was enhanced by LP‐KSFY06 and that the combination resulted in increased weight, moisture content, and particle number of feces. The first black stool defecation time was decreased from 182 min to 87 min, which clearly indicates that defecating difficulty was alleviated in constipated mice. The synergic intervention of LP‐KSFY06 and geniposide (LP + G) assisted in maintaining the body weight of constipated mice. The LP + G intervention significantly increased serum levels of motilin (MTL, 167.8 pg/ml), acetylcholinesterase (AChE, 45.3 pg/ml), substance P (SP, 61.0 pg/ml), vasoactive intestinal peptide (VIP, 70.5 pg/ml), endothelin‐1 (ET‐1, 16.1 pg/ml), and gastrin (73.0 pg/ml) and remarkably decreased somatostatin (SS, 35.2 pg/ml) when compared to those indexes in the LP‐KSFY06 group and geniposide group. The LP + G treatment also significantly increased the mRNA expression of cluster of differentiation 117 (c‐Kit), stem cell factor (SCF), glial cell‐derived neurotrophic factor (GDNF), and remarkably downregulated the expression of inducible nitric oxide synthase (iNOS), transient receptor potential vanilloid‐1 (TRPV1), and cyclooxygenase‐2 (COX‐2). The experimental results showed that the combination treatment has the strongest prevention effect against constipation, and LP‐KSFY06 promotes the ability of geniposide to prevent constipation. Therefore, LP‐KSFY06 is a potential probiotic strain with the capacity to prevent montmorillonite‐induced constipation.

## Graphical abstract

Combined effects of *Lactobacillus plantarum* KSFY06 and geniposide could inhibit the montmorillonite‐induced constipation in Kunming mice.

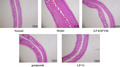

## 1 INTRODUCTION

Constipation is a clinical manifestation of digestive system disorders and is characterized by the formation of hard feces that are difficult to excrete. The prevalence of constipation ranges from 2% to 30%, especially in children and geriatric populations (Chen, Chao, & Pan, 2020; Zhao et al., 2019). Constipation can be divided into primary and secondary constipation, with primary constipation including three types (i.e., normal‐transit constipation, slow‐transit constipation, and defecatory disorders) (Lembo & Camilleri, 2003). Unhealthy eating and excretion habits, lack of exercise, neurological disorders, or irregular metabolic conditions can trigger constipation, although the exact pathogenesis of constipation is still unclear (Schuster, Kosar, & Kamrul, 2015). The duration of stools retained in the large intestine is prolonged when constipation occurs, and fecal moisture is subsequently absorbed, resulting in dry feces that inhibit the movement of the bowel and induce intestinal imbalance and bowel pain (Chen, Wu, Liao, & Yang, 2010; Khalif, Quigley, Konovitch, & Maximova, 2005). Constipation is a risk factor for irritable bowel syndrome and colorectal cancer, as well as other gastrointestinal disorders (Okawa, Fukudo, & Sanada, 2019; Shimotoyodome, Meguro, Hase, Tokimitsu, & Sakata, 2000). Administration of laxatives and prokinetic agents (motilin agonists, 5‐hydroxytryptamine modulators, opioid antagonists, and chloride‐channel activators) are common clinical treatments for constipation (Shin et al., 2014). Long‐term intake of anticonstipation agents, however, can cause drug dependence and is economically unfeasible due to direct and indirect health care expenses (Crowell, Harris, Lunsford, & Dibaise, 2009). Laxatives can also cause side effects such as diarrhea, upset stomach, stomach cramping, and vomiting.

Constipation is generally not regarded as a disease. It is a multifactorial disorder that can be managed by exercise and consumption of a healthy diet and additional water (Webster, Tummala, Diva, & Lappalainen, 2016). Nutritional therapy for constipation consists of consumption of fruits, vegetables, whole grains, and cereals, which are rich in dietary fiber, and can increase fecal mass and accelerate the small intestine passage, which subsequently reduces colonic transit time (Karabudak, Koksal, & Macit, 2019; Wang, Sun, Zhou, & Zhao, 2015). Moreover, increasing numbers of studies indicate that probiotics (i.e., *Lactobacillus* and *Bifidobacterium*) decrease whole‐gut transit time and increase defecation frequency by increasing intestinal motility, as well as softening fecal consistency (Eskesen et al., 2015; Wojtyniak & Szajewska, 2017).

Probiotics are defined as living microorganisms, and only when administered in adequate amounts can they confer beneficial health effects upon the host (FAO/WHO, 2006). Lactic acid bacteria (LAB) are an important group of probiotics and include *Lactobacillus* spp., *Bifidobacterium* spp., and *Enterococcus* spp., which are widely used as food supplements. LAB directly or indirectly act on the large intestine and other organs by influencing the intestinal flora, modulating intestinal permeability and immunological parameters, and producing regulatory or bioactive metabolites (Markowiak & Slizewska, 2017).

Geniposide is an iridoid glycoside extracted from the fruit of *Gardenia jasminoides* that is widely used in Asian countries. As a natural bioactive constituent, it has been proven that geniposide is a curative substance for diseases of the digestive system (Qian, Suo, Yi, Li, & Zhao, 2017). *Lactobacillus* strains also can effectively alleviate constipation (Chen et al., 2020; Zhao et al., 2019). Therefore, this study evaluated the potential synergic effect of the combination of *Lactobacillus plantarum* KSFY06 (LP‐KSFY06) and geniposide. We attempted to determine if the bacterial strain has the ability to increase the effectiveness of geniposide, which provides a theoretical basis for further in‐depth research, application, and industrial development.

## 2 MATERIALS AND METHODS

### 2.1 Experimental strain

*Lactobacillus plantarum* KSFY06 (LP‐KSFY06) was isolated from naturally fermented yogurt from Xinjiang, China. It was identified using Basic Local Alignment Search Tool (BLAST) at the National Center for Biotechnology Information (NCBI) website.

### 2.2 Design of a mouse constipation model

Fifty male, 6‐week‐old Kunming (KM) mice were purchased from the Experimental Animal Center of Chongqing Medical University (Chongqing, China). They were acclimatized for 1 week at room temperature of 25 ± 2°C with relative humidity 55 ± 5% and a 12 hr light/12 hr dark cycle, with diet and water ad libitum. Then, the mice were randomly divided into 5 groups (*n* = 10/group): (a) normal, (b) model, (c) LP‐KSFY06, (d) geniposide, and (e) LP‐KSFY06 and geniposide (LP + G). All groups were fed a standard rodent AIN‐93G chow diet (Jiangsu Medicine Ltd.). The normal and model groups received a normal diet for 9 days, and the treatment groups received a normal diet with LP‐KSFY06 (0.5 × 10^7^ colony‐forming units (CFU)/kg body weight), geniposide (50 mg/kg body weight), and LP + G (LP‐KSFY06 1 × 10^7^ CFU/kg body weight and 50 mg/kg body weight). Montmorillonite powder suspension (30 mg/kg body weight) was orally administered to the model and intervention groups from d 7 to d 9 to induce constipation. Body weight (BW), stool weight, and moisture were recorded. This study was approved by the Ethics Committee of Chongqing Medical University (SCXK 2017‐0001).

### 2.3 Gastrointestinal transit ratio and defecation time

All groups were fasted for 24 hr with free access to water. The model and intervention groups received an oral administration of 0.2 ml of 10% activated carbon that was dissolved in 10% gum arabic, while the normal group received oral administration of 10% gum arabic. Half of the mice from each group were killed by CO_2_ following 30 min of treatment, and small intestines were collected. The gastrointestinal transit ratio for each mouse was calculated as:

$$\text{gastrointestinal transit ratio}\;\left(\%\right)=\text{distance the activated carbon traveled}/\text{total length of the small intestine}\times 100$$

The remaining 5 mice from each group were used to measure the defecation time of the first black stool.

### 2.4 Morphology of small intestinal villi from mice

All mice were killed using CO_2_, and the blood, colon tissue, and small intestine were collected. The small intestine of each mouse was cut into lengths of 2 cm and then fixed in 10% formalin. After dehydrating for 48 hr, transparency treatment, wax immersion, embedding, sectioning, and hematoxylin and eosin (H&E) staining were performed to observe the morphological changes of these tissues under optical microscopy (BX43, Olympus) (Chen et al., 2019).

### 2.5 Determination of serum gastrointestinal motility‐related biomarkers

The blood was centrifuged (1,500 *g*, 4°C, 10 min) to obtain serum, which was collected in Eppendorf tubes and stored at −80°C pending analysis. Then, serum levels of motilin (MTL), acetylcholinesterase (AChE), substance P (SP), vasoactive intestinal peptide (VIP), gastrin, somatostatin (SS), and endothelin‐1 (ET‐1) were measured by corresponding kits (Beijing Puer Weiye Biotechnology Co. Ltd.).

### 2.6 Total RNA extraction and quantitative PCR assay

The small intestine tissue from each mouse was washed with normal saline and homogenized. Total RNA was extracted with TRIzol reagent according to the manufacturer's protocol (Thermo Fisher Scientific). The purity and concentration of the extracted total RNA were determined by ultra‐micro spectrophotometry (Nano‐100, All for Life Science), and then, the RNA was diluted to 1 μg/μl. Total RNA samples were used as templates to generate cDNAs by reverse transcription (Thermo Fisher Scientific). Then, cDNAs (2 μl) were mixed with 2× SYBR Premix Ex Taq II (10 μl), 50× ROX reference dye (0.4 μl), total primer (2 μl, 10 μmol/L, Table 1), and distilled H_2_O (5.6 μl). The PCR was performed in an automatic thermocycler (QuantStudio 6 Flex PCR, Life Technologies) with the following parameters: 95°C for 60 s; 40 cycles of 94°C for 30 s, 58°C for 30 s, and 72°C for 50 s; 75°C for 10 min. GAPDH was selected as an internal reference, and the relative expression of cluster of differentiation 117 (*c‐Kit*), stem cell factor (*SCF*) glial cell‐derived neurotrophic factor (*GDNF*), inducible nitric oxide synthase (*iNOS*), transient receptor potential vanilloid‐1 (*TRPV1*) and cyclooxygenase‐2 (*COX‐2*) mRNA was calculated using the 2^−ΔΔCt^ formula (Zhao et al., 2019).

**TABLE 1:** Stool status of mice during the experiment

Item	Stool weight (g)		Stool water content (%)	
	Day 1–6	Day 7–9	Day 1–6	Day 7–9
Normal	1.01 ± 0.09^a^	1.02 ± 0.05^a^	48 ± 2.9^a^	48 ± 2.9^a^
Model	0.99 ± 0.05^a^	0.49 ± 0.03^c^	47 ± 2.5^a^	20 ± 2.1^e^
LP‐KSFY06	1.02 ± 0.08^a^	0.58 ± 0.04^c^	47 ± 2.6^a^	26 ± 1.0^d^
Geniposide	1.01 ± 0.03^a^	0.73 ± 0.05^b^	48 ± 3.6^a^	32 ± 4.2^c^
LP + G	1.00 ± 0.04^a^	0.83 ± 0.08^b^	47 ± 2.8^a^	38 ± 2.0^b^

Note

Values presented are the mean ± standard deviation (*n* = 10/group).

Day 1–6: treatment period without induction of constipation; Day 7–9: treatment period with induction of constipation.

LP‐KSFY06: mice treated with 0.5 × 10^7^ CFU/kg of *Lactobacillus plantarum* KSFY06; Geniposide: mice treated with 50 mg/kg body weight of geniposide; LP + G: mice treated with 1.0 × 10^7^ CFU/kg of *Lactobacillus plantarum* KSFY06 and 50 mg/kg body weight of geniposide.

^a‐e^In the same column means significant differences (*p* < .05) according to Duncan's multiple range test.

### 2.7 Statistical analysis

In this study, the experiments were carried out in triplicate, and the mean values were obtained. All the data, presented as the mean ± standard deviation, were analyzed by SPSS 22 software (SPSS Inc., Chicago, IL, USA). Significant differences among groups, presented by *p* < .05, were analyzed by one‐way analysis of variance (ANOVA). All figures were drawn using Origin 8.1 software.

## 3 RESULTS

### 3.1 Body weights of mice

**FIGURE 1:**
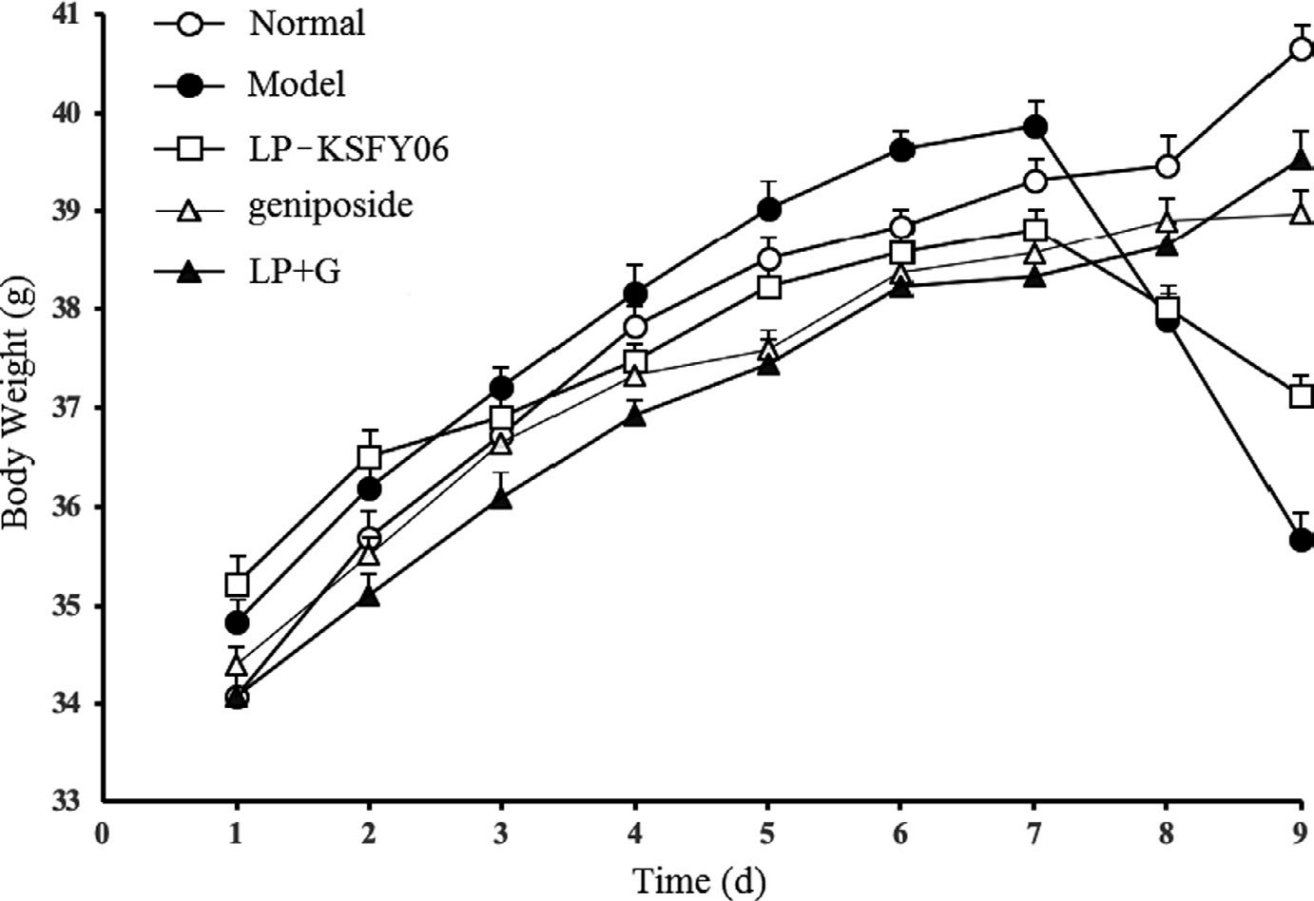
Body weight of mice during the experiment. LP‐KSFY06: mice treated with 0.5 × 10^7^ CFU/kg of *Lactobacillus plantarum* KSFY06; Geniposide: mice treated with 50 mg/kg body weight of geniposide; LP + G: mice treated with 1.0 × 10^7^ CFU/kg of *Lactobacillus plantarum* KSFY06 and 50 mg/kg body weight of geniposide

The changes in BW shown in Figure 1 illustrated that gradual growth of mice in all groups occurred during the first six days. From day 7, the BWs of the model group were significantly decreased due to constipation induced by montmorillonite clay. The LP‐KSFY06 group exhibited the same tendency of BW. However, the treatment consisting of geniposide or LP + G inhibited weight loss compared with the model group (*p* < .05), and BW steadily increased.

### 3.2 Effect of LP‐KSFY06 and geniposide on fecal parameters

The fecal parameters shown in Table 1 indicate that montmorillonite significantly decreased the weight and water content of stool from 0.99 g to 0.49 g and 47% to 20%, respectively (*p* < .05). LP‐KSFY06 treatment enhanced the stool moisture of the constipated mice (*p* < .05), while there was no effect on stool weight (*p* > .05). The geniposide or LP + G intervention significantly increased the stool weight and water content (*p* < .05).

### 3.3 Gastrointestinal transit capability in constipation with different treatments

As shown in Table 2, montmorillonite did not influence the length of the small intestine, nor did the LP‐KSFY06 and geniposide. However, constipation without treatment significantly decreased the gastrointestinal transit capability, with the activated carbon propulsive rate reduced to 39.3% (*p* < .05), and increased the first black stool defecation time to 182 min (*p* < .05). Both LP‐KSFY06 and geniposide separately enhanced the gastrointestinal transit capability, and the combination of LP‐KSFY06 and geniposide (LP + G) exhibited more effective regulation.

**TABLE 2:** Gastrointestinal transit capability and time to first black stool defection (*n* = 5)

Group	Length of small intestine (cm)	Activated carbon propulsive rate (%)	Time to first black stool defecation (min)
Normal	50.4 ± 0.9^a^	100.0 ± 0.0^a^	67 ± 18^e^
Model	49.8 ± 1.2^a^	39.3 ± 1.8^e^	182 ± 20^a^
LP‐KSFY06	50.2 ± 1.0^a^	50.1 ± 1.6^d^	133 ± 20^b^
Geniposide	50.6 ± 1.0^a^	69.1 ± 4.4^c^	118 ± 19^c^
LP + G	51.1 ± 1.2^a^	80.3 ± 2.0^b^	87 ± 21^d^

Note

Values presented are the mean ± standard deviation (*n* = 5/group).

Day 1–6: treatment period without induction of constipation; Day 7–9: treatment period with induction of constipation.

LP‐KSFY06: mice treated with 0.5 × 10^7^ CFU/kg of *Lactobacillus plantarum* KSFY06; Geniposide: mice treated with 50 mg/kg body weight of geniposide; LP + G: mice treated with 1.0 × 10^7^ CFU/kg of *Lactobacillus plantarum* KSFY06 and 50 mg/kg body weight of geniposide.

^a‐e^In the same column means significant differences (*p* < .05) according to Duncan's multiple range test.

### 3.4 Morphology of small intestinal villi from mice

**FIGURE 2:**
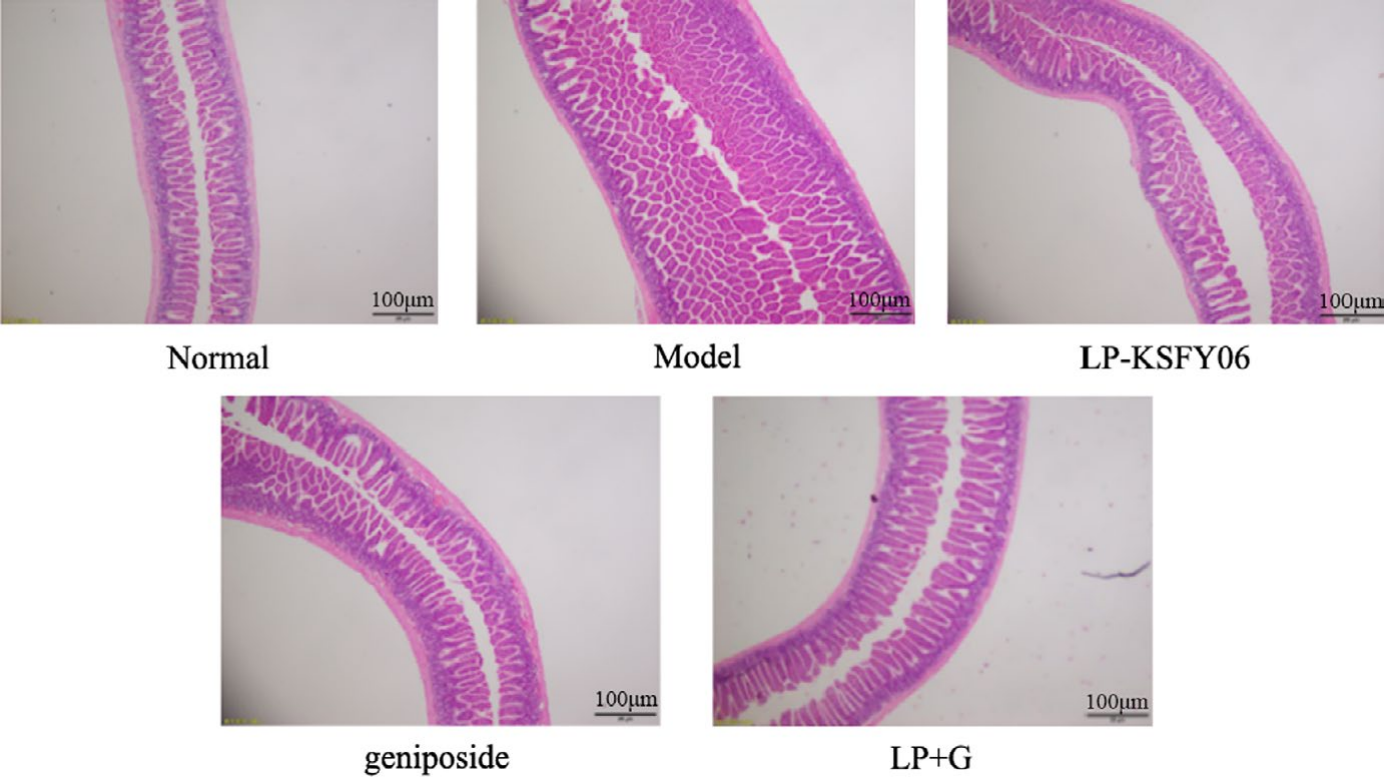
Hematoxylin–eosin (H&E) pathological observation of small intestinal villi from mice. Magnification 40×. LP‐KSFY06: mice treated with 0.5 × 10^7^ CFU/kg of *Lactobacillus plantarum* KSFY06; Geniposide: mice treated with 50 mg/kg body weight of geniposide; LP + G: mice treated with 1.0 × 10^7^ CFU/kg of *Lactobacillus plantarum* KSFY06 and 50 mg/kg body weight of geniposide

Morphological section examination of small intestinal villi (Figure 2) in the model group revealed noticeable edema of the lamina propria, and irregular arrangement of the intestinal villi. All treatments significantly dissipated the edema of the lamina propria and repaired the intestinal villi. Moreover, the pathological changes of the intestinal villi in the LP + G group were recovered to a status similar to that in the normal group.

### 3.5 Determination of serum gastrointestinal motility‐related biomarkers

The serum gastrointestinal motility‐related biomarkers were determined and are shown in Table 3. The results indicate that montmorillonite (model group) significantly decreased the levels of MTL, VIP, AchE, gastrin, and SP (*p* < .05), while it remarkably increased the level of ET‐1 and SS (*p* < .05). With the intervention of LP‐KSFY06, geniposide, or LP + G, these influenced biomarkers were attenuated. A more optimal regulatory effect was observed after LP + G treatment in constipated mice, and indexes of serum gastrointestinal motility were similar to those of the normal group.

**TABLE 3:** Effect of *Lactobacillus plantarum* KSFY06 and geniposide on serum level (pg/mL) of gastrointestinal motility‐related biomarkers in mice with montmorillonite power‐induced constipation (*n* = 10)

Group	MTL	ET‐1	VIP	AchE	Gastrin	SP	SS
Normal	192.5 ± 13.5^a^	13.3 ± 1.6^e^	80.4 ± 7.7^a^	50.9 ± 15.7^a^	90.6 ± 5.9^a^	71.5 ± 4.3^a^	28.3 ± 1.6^d^
Model	92.7 ± 13.7^e^	31.0 ± 6.4^a^	38.4 ± 3.1^e^	16.9 ± 5.8^d^	41.5 ± 2.7^e^	33.6 ± 2.2^e^	61.5 ± 6.3^a^
LP‐KSFY06	127.7 ± 8.9^d^	24.8 ± 2.7^b^	49.1 ± 2.8^d^	28.5 ± 3.0^c^	45.6 ± 4.3^d^	43.5 ± 1.8^d^	48.1 ± 1.6^b^
Geniposide	143.6 ± 6.9^c^	18.8 ± 1.5^c^	54.6 ± 4.8^c^	37.6 ± 5.0^b^	65.2 ± 3.8^c^	54.3 ± 4.6^c^	41.4 ± 1.9^c^
LP + G	167.8 ± 14.8^b^	16.1 ± 3.2^d^	70.5 ± 4.9^b^	45.3 ± 6.1^a^	73.0 ± 4.6^b^	61.0 ± 4.8^b^	35.2 ± 2.9^d^

Note

Values presented are the mean ± standard deviation (*n* = 10/group).

Day 1–6: treatment period without induction of constipation; Day 7–9: treatment period with induction of constipation.

LP‐KSFY06: mice treated with 0.5 × 10^7^ CFU/kg of *Lactobacillus plantarum* KSFY06; Geniposide: mice treated with 50 mg/kg body weight of geniposide; LP + G: mice treated with 1.0 × 10^7^ CFU/kg of *Lactobacillus plantarum* KSFY06 and 50 mg/kg body weight of geniposide.

Abbreviations: AChE, acetylcholinesterase; ET‐1, endothelin‐1; MTL, motilin; SP, substance P; SS, somatostatin; VIP, vasoactive intestinal peptide.

^a‐e^In the same column means significant differences (*p* < .05) according to Duncan's multiple range test.

### 3.6 RNA expression in the mice small intestine

**FIGURE 3:**
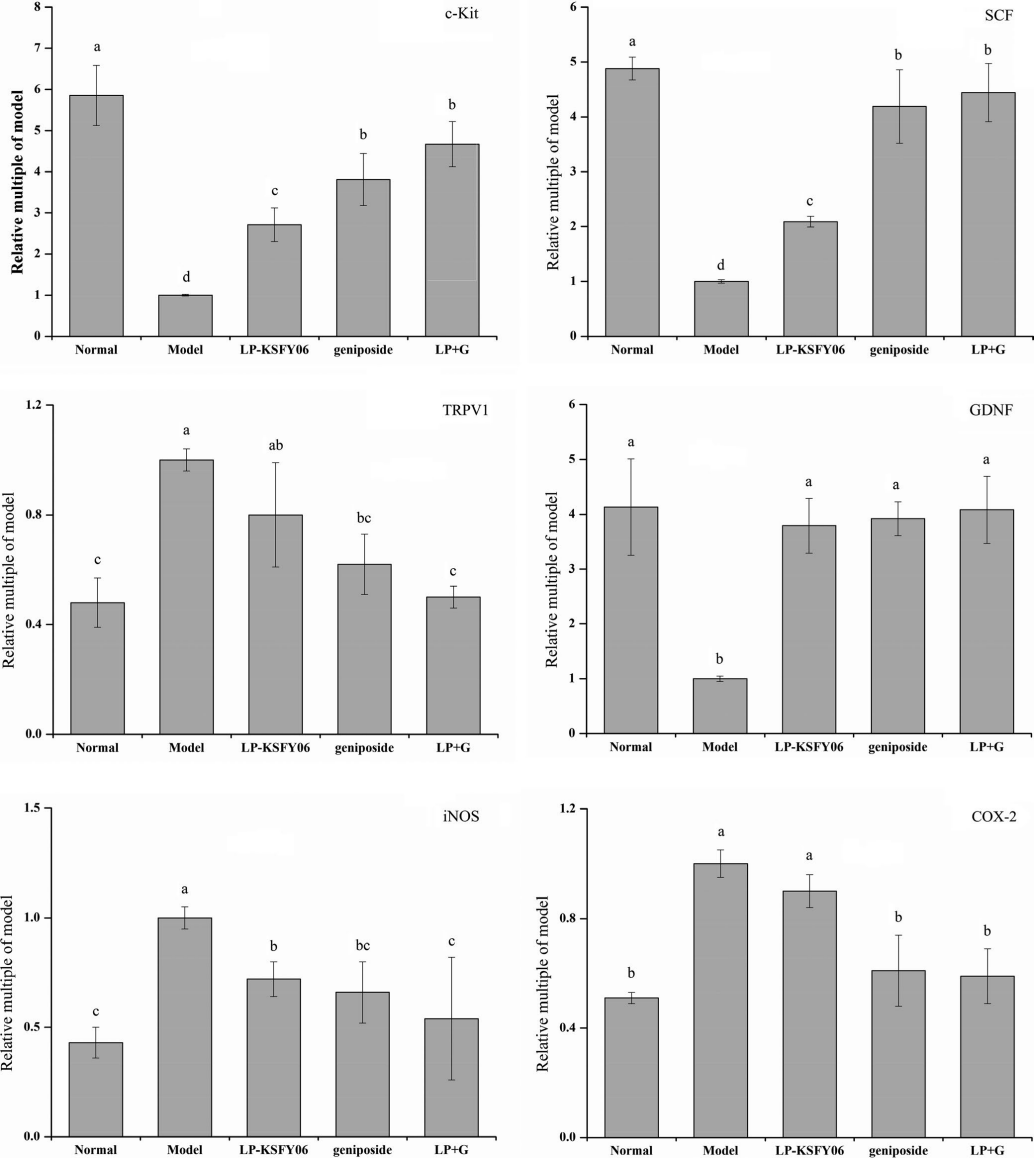
The cluster of differentiation 117 (c‐Kit), stem cell factor (SCF), glial cell‐derived neurotrophic factor (GDNF), inducible nitric oxide synthase (iNOS), transient receptor potential vanilloid‐1 (TRPV1), cyclooxygenase‐2 (COX‐2) mRNA expression in small intestine of mice. ^a–d^The different letters mean that there are significant differences (*p* < .05) between every two groups between every two groups according to Duncan's multiple range test. LP‐KSFY06: mice treated with 0.5 × 10^7^ CFU/kg of *Lactobacillus plantarum* KSFY06; Geniposide: mice treated with 50 mg/kg body weight of geniposide; LP + G: mice treated with 1.0 × 10^7^ CFU/kg of *Lactobacillus plantarum* KSFY06 and 50 mg/kg body weight of geniposide

Compared with the normal group, the expression of *c‐Kit*, *SCF*, and *GDNF* mRNA in the model group was upregulated, and *TRPV1*, *iNOS*, and *COX‐2* mRNA expression was downregulated (Figure 3). After intervention with LP‐KSFY06, geniposide, or LP + G, the changes induced by constipation were significantly attenuated in all the experimental groups (all *p* < .05), except for LP‐KSFY06 alone and its effects on *TRPV1* and *COX‐2*. The efficacy of LP + G was superior to that of sole treatments with LP‐KSFY06 or geniposide. Moreover, LP‐KSFY06 improved the regulatory effect of geniposide on mRNA expression of some marker molecules in montmorillonite‐induced constipation.

## 4 DISCUSSION

Constipation is a common problem in humans, and current treatments do not provide relief of symptoms. Studies have shown that constipation is associated with gut microbiota dysbiosis, and decreased abundance of *Lactobacillus*, *Bifidobacteria*, *Prevotella*, and *Bacteroides* might be a potential cause or a consequence of altered gut motility (Attaluri, Jackson, Valestin, & Rao, 2010; Wojtyniak, Horvath, Dziechciarz, & Szajewska, 2017). With the recognition of the gut microbiota, probiotics can be used to modify health and disease, even though the exact mechanisms remain unclear. LAB probiotics are widely used in the food industry and have gradually developed into relatively indispensable functional foods that are eaten on a daily basis (Filanniono, Cagno, & Gobbetti, 2018; Venegas‐Ortega, Flores‐Gallegos, Martinez‐Hernandez, Aguilar, & Nevarez‐Moorillion, 2019). Studies have shown that LAB can alter the sensation and motility of the gut (Quigley, 2007; Rousseaux et al., 2007), soften the stool, and change secretion, as well as produce lactic acid and/or short‐chain fatty acids that can lower the intraluminal pH and promote defecation (Salminen & Salminen, 1997; Waller et al., 2011).

Patients with constipation usually exhibit difficulty with gastrointestinal peristalsis and voiding (Dimidi, Christodoulides, Fragkos, Scott, & Whelan, 2014; Suo et al., 2014), due to lower water content in the stool. In this study, montmorillonite‐induced constipation resulted in significantly decreased weight and moisture of stool, and decreased activated carbon propulsive rate that prolonged the time to first black stool defecation. Oral administration of LP‐KSFY06 (Table 1) increased gastrointestinal peristalsis (Table 2) and relieved intestinal damage. Moreover, LP‐KSFY06 promoted the interventional effects of geniposide.

Enteric nervous parameters secreted by the enteric nerve network in the gastrointestinal tract act as neuromodulators and neurotransmitters, promoting intestinal peristalsis and transportation of contents (Qian et al., 2015; Ramos et al., 2017). MLT, SP, and VIP are excitatory peptides, and AchE is an excitatory enzyme neurotransmitter, while ET‐1 and SS are inhibitory peptide neurotransmitters (Nassif et al., 1995; O'Dea, Brookes, & Wattchow, 2010; Wang, Hu, Xu, et al., 2017; Wang, Hu, Yan, et al., 2017). Gastrin, mainly secreted by the gastric sinus, promotes gastrointestinal motility, and the secretion is inhibited by SS (Jiang et al., 2020). In the current study, combination treatment with LP‐KSFY06 and geniposide (LP + G group) significantly enhanced levels of serum MTL, VIP, gastrin, AchE, and SP and inhibited serum ET‐1 and SS, of which the effects were remarkably superior to those of treatments with LP‐KSFY06 or geniposide alone. The serum levels of these biomarkers in the combination treatment group were similar to those in the normal group.

Apart from direct regulation of these neurotransmitters, LP‐KSFY06 also affects RNA expression in the small intestine. The interstitial cells of Cajal (ICC) are nerve‐like cells at the ends of motor neurons that maintained by c‐Kit, which promotes intestinal motility in the gastrointestinal system (Bansil & Turner, 2018; Lee, Park, Kamm, & Talbot, 2005; Yu, Crowell, Tihan, & Lacy, 2002). Low levels of ICC can be observed in patients with constipation (Sabri, Barksdale, & Lorenzo, 2003; Su et al., 2019). LAB could improve constipation by upregulating the expression of *c‐Kit* mRNA (Chen et al., 2020). SCF is a ligand for c‐Kit and necessary for the appropriate development and survival of ICC in the intestine (Wedel et al., 2002). In previous studies, the mRNA expression of *c‐Kit* and *SCF* in constipated mice or patients with constipation was decreased compared with that in normal mice and healthy individuals (Lee et al., 2005; Li et al., 2010). Our results also showed that there were lower mRNA levels of *c‐Kit* and *SCF* in model mice compared with those in the normal group. Additionally, combination LP‐KSFY06 and geniposide treatment enhanced the expression of *c‐Kit* and *SCF* mRNA (Figure 3).

Transient receptor potential cation channel subfamily V member 1 (TRPV1), a member of the TRPV group of transient receptor potential family of ion channels, is associated with the release of SP and closely related to defecation (Geppetti & Trevisani, 2004; Peng & Li, 2010), and its overexpression might indicate intestinal injury (Su et al., 2019). Treatment with LP‐KSFY06 reduced the increased expression level of *TRPV1* to some degree, while the combination of LP‐KSFY06 and geniposide nearly eliminated the increased expression of *TRPV1* in a manner similar to that in normal mice.

GDNF is a protein distributed in the gastrointestinal tract that modulates the growth and development of nerve cells (Allen, Watson, Shoemark, Barua, & Patel, 2013), repairs damaged nerve fibers, as well as promotes intestinal epithelial cell proliferation (Kalff, Schraut, Billiar, Simmons, & Bauer, 2000) and repairs the damaged intestinal tract (Su et al., 2019). As shown in Figure 3, the mRNA expression of *GDNF* in model mice was significantly inhibited by montmorillonite‐induced constipation. The inhibitory effects were totally negated, and there were no differences among mice treated with LP‐KSFY06, geniposide, or the combination.

Nitric oxide synthase (NOS) is the key to the produce endogenous NO from the esophagus to the anal sphincter. Activated inducible nitric oxide synthase (iNOS) hardly expresses in intestinal smooth muscle cells under normal circumstances which directly modulates intestinal dysmotility (Ma et al., 2019). However, a mass of NO derived from iNOS under inflammatory reaction will suppress the contractility of intestinal smooth muscle (Moojen et al., 1999; Toda & Okamura, 2016). Moreover, prostaglandin released by cyclooxygenase‐2 (COX‐2) also inhibits the intestinal smooth muscle contractility (Schwarz et al., 2001; Su et al., 2019). Inhibition of the expression of either *iNOS* or *COX‐2* can significantly enhance the motility of the small intestine (Wen et al., 2006). In the current study, the mRNA expression of *iNOS* and *COX‐2* significantly increased by 1.33‐fold and 0.95‐fold when compared to that in the normal group (Figure 3). After treatment with the combination of LP‐KSFY06 and geniposide, the mRNA expression of *iNOS* and *COX‐2* was significantly decreased. However, LP‐KSFY06 showed no effect on regulating the expression of *COX‐2* mRNA.

In this study, oral administration of LP‐KSFY06 and geniposide attenuated the montmorillonite‐induced constipation in mice. In addition, *L. plantarum* KSFY06 enhanced the effect of geniposide on constipation. The combination of KSFY06 and geniposide effectively increased stool weight and water content and increased the activated carbon propulsive rate, shortened the defecation time of the first black stool, and repaired intestinal villi. In addition, serum levels of MTL, VIP, AchE, gastrin, and SP were increased, and ET‐1 and SS were decreased to a greater degree after LP + G treatment compared with LP‐KSFY06‐ or geniposide‐treated mice. LP + G‐treated mice also demonstrated significantly increased mRNA expression levels of *c‐Kit*, *SCF*, and *GDNF* and decreased *TRPV1*, *iNOS*, and *COX‐2*. These results suggest that LP‐KSFY06 promoted geniposide attenuation of montmorillonite‐induced constipation in mice, and LP‐KSFY06 could be a potential agent that can be used for prevention or healing of constipation.

## 5 CONCLUSIONS

LP‐KSFY06 alleviated symptoms and reverted intestinal villi changes associated with constipation. Even though there were no differences in *c‐Kit*, *SCF*, *GDNF*, *TRPV1*, *iNOS*, and *COX‐2* mRNA expression between the genisoside and LP + G groups, LP‐KSFY06 improved the regulatory effect of geniposide on gastrointestinal motility‐related biomarkers in constipated mice. This study indicated that *L. plantarum* KSFY06 may be an effective probiotic candidate with the ability to mitigate the adverse effects of constipation. The mechanism used by LP‐KSFY06 to promote geniposide's action will be more closely examined, and clinical trials will be carried out to verify their effectiveness in humans.

## CONFLICT OF INTEREST

There are no conflicts of interest in this paper.

## Data Availability

No data were used to support this study.
